# Polymorphisms of DNA Repair Genes in Endometrial Cancer

**DOI:** 10.1007/s12253-012-9537-5

**Published:** 2012-04-28

**Authors:** Anna Sobczuk, Tomasz Poplawski, Janusz Blasiak

**Affiliations:** 1 Department of Gynaecology and Obstetrics, Medical University of Lodz, Lodz, Poland; 2 Gynaecology and Oncology Clinic, Polish Mother’s Memorial Institute, Lodz, Poland; 3 Department of Molecular Genetics, University of Lodz, Lodz, Poland

**Keywords:** XRCC1, ERCC2, hOGG1, Endometrial cancer, RFLP-PCR, BER, NER

## Abstract

Endometrial cancer belongs to the commonest malignancy in females. Its development may be associated with the high exposure of endometrium to exo- and endogenous estrogens. Estrogens produce DNA bulky adducts and oxidative base damages which are removed in nucleotide excision repair (NER) and base excision repair (BER) pathways. The reaction of endometrial cells to DNA damage may be crucial for their susceptibility to cancer transformation. This reaction is executed mainly by DNA repair, which can be modulated by the variability in the genes encoding DNA repair proteins. In this report we genotyped 4 polymorphisms of 3 DNA repair genes in 94 endometrial cancer patients and 114 age-matched cancer-free women using RFLP-PCR. The following polymorphisms were studied: p.Arg194Trp, p.Arg399Gln of the *XRCC1* gene, p.Ser326Cys of the *hOGG1* gene and p.Lys751Gln of the *ERCC2* gene. We found an association between the *ERCC2* 751Gln variant and endometrial cancer occurrence (OR 3.95; 95 % CI 1.88–8.31). Gene-gene interaction between the *ERCC2* 751Gln and *XRCC1* 194Trp variants also increased the risk of endometrial cancer (OR 4.41; 95 % CI 2.01–9.67). The risk in the carriers of the *ERCC2* 751Gln variant was increased by a positive cancer history in first degree relatives (OR 4.97; 95 % CI 1.98–12.48). The risk of endometrial cancer was not alter by polymorphism p.Ser326Cys of the hOGG1 gene. The 751 Lys/Gln polymorphism of the *ERCC2* gene may be linked with endometrial cancer occurrence and its effect can be potentiated by variants of the *XRCC1* gene or first degree relatives positive cancer history.

## Introduction

Endometrial carcinoma (EC) is the most common tumor of the female genital tract in the Western world [1]. The great majority of EC cases are type I (estrogen-related), frequently showing microsatellite instability and mutations in the *PTEN, PIK3CA, K-Ras* and β*-catenin* genes. These mutations may reflect the genomic instability which is most common symptom of the cancer cells [2, 3]. This instability may be caused by a continuous exposure to genotoxic stress, including that evoked by estrogens, which can induce bulky DNA adducts and minor modifications to the DNA bases [4]. These lesions are removed by nucleotide and base excision repair (NER and BER), respectively. NER includes recognition of DNA damage by the RAD23B-XPC complex, followed by binding of the XPA/RPA dimer to the lesion. XPA is an important factor for accurate positioning of the ERCC1-ERCC4 (XPF) endonuclease. Two helicases ERCC3 (XPB) and ERCC2 (XPD) are responsible for unwinding the DNA helix, and the ERCC5 (XPG) and ERCC1-ERCC4 nucleases excise a single stranded DNA fragment containing the lesion. The remained gap is filled by DNA polymerase δ/ε and DNA ligase I using the intact strand as a template. The base excision repair (BER) pathway corrects most base modifications caused by reactive oxygen species (ROS). A damaged base is recognized by a specific glycosylase, which cleaves the bond between the base and sugar, creating an abasic site, which is cleaved by an endonuclease. Resulting gap is filled by polβ and the remaining nick is sealed by DNA ligase LIG1 or LIG3 complexed with XRCC1.

Because NER and BER are involved in removing a substantial number of DNA damages, which can contribute to the genome instability, it is reasonable to check whether variability in the genes coding for BER and NER products may be associated with EC. In the present work we searched for an association between EC and the variants of single nucleotide polymorphisms (SNPs) of the BER/NER genes: *ERCC2, OGG1* and *XRCC1*. We studied 4 SNPs occurring in 3 BER and NER genes: p.Arg194Trp, p.Arg399Gln of the *XRCC1* gene, p.Ser326Cys of the hOGG1 gene and p.Lys751Gln of the *ERCC2* gene (rs1799782, rs25487, rs1052133 and rs13181 respectively). These polymorphisms have been correlated with various tumors, including lung, breast and skin cancers [5–13], but little is known about their association with EC.

## Materials and Methods

### Patients

Blood was obtained from 94 women (median age 48 years and median BMI 28) with EC treated in 2004–2006 at the Polish Mother’s Memorial Hospital (Lodz, Poland). All patients had histologically confirmed EC and agreed to complete a risk factor questionnaire. The characteristics of the subjects enrolled in this study are presented in Table 1. Control samples consisted of DNA extracted from blood cells from age-matched 114 cancer-free women. The study was approved by the Local Ethic Committee and each patient gave a written consent.

**Table 1:** Characteristics of the study population

Characteristics	Cases (*n* = 94)	Controls (*n* = 114)
Age (y)		
Mean	61	55
Min	43	45
Max	83	84
Education		
Elementary school	21	22
Secondary technical school	15	15
High school	38	50
More than high school	20	27
No. of birds		
0	14	19
1	28	22
>1	52	73
Body mass index		
<19	0	0
18–25	25	37
26–29	40	50
>30	29	27
First menarche		
Before 11 years	5	10
12–13 years	35	56
14–15 years	43	30
After 16 years	9	11
Missing	2	7
Hypertension		
	51	43
HRT		
Yes	15	31
No	79	74
Missing	0	9
Smoking		
No	64	73
Past or Current	26	36
Missing	4	5
Alcohol consumption		
Yes	49	69
No	43	39
Missing	4	6
Family cancer		
Yes	29	19
No	63	84
Missing	4	11
FIGO stage		
I	71	
II	8	
III	13	
IV	2	
FIGO grade		
G1	41	
G2	28	
G3	25	

### Genotype Determination

Genomic DNA was prepared using GeneMatrix Blood DNA purification Kit (EURx, Gdansk, Poland) according to the manufacturer instruction. Genotypes were determined by PCR-RFLP (polymerase chain reaction-restriction fragment length polymorphism). Genome regions that include studied polymorphisms were amplified by PCR using primers listed in Table 2. The PCR reaction (total volume 25 μl) was launched with a mixture containing 100 ng genomic DNA, 5 mM dNTPs, 5 pmol each primer and 1 U Taq DNA polymerase (Biotools, Madrid, Spain) which was added into PCR buffer containing 10 mM Tris–HCl, 1.5 μM MgCl_2_ and 50 mM KCl. PCR conditions were as follows: initial denaturation step at 95 °C for 5 min, 30 cycles at 95 °C for 30 s and 30 s at the 62 °C annealing temperature, and at 72 °C for 30 s. The final extension step was performed at 72 °C for 5 min. The PCR was carried out in a MJ Research, INC thermal cycler, model PTC-100 (Waltham, MA, USA). Following PCR, 20 ml aliquots were removed and subjected to restriction digestion with *Pvu*II (for codon 194), *Bcn*I (for codon 399), *Sat*I (for codon 326) or *Pst*I (for codon 751). All restriction enzymes were from Fermentas, Vilnius, Lithuania). The digested products were resolved on a 8 % acrylamide gel and stained with 0.5 μg/ml ethidium bromide. The cleavage of the *XRCC1* fragment with *Pvu*II produced bands of 292/174/21, 313/292/174/21 and 313/174 bp corresponding to the Arg/Arg, Arg/Trp and Trp/Trp genotypes, respectively. The *Bcn*I restrictase having acted on the same fragment produced bands of 159/89, 248/159/89 and 248 bp corresponding to the 399 Arg/Arg, Arg/Gln and Gln/Gln genotypes, respectively. The *Sat*I restriction enzyme yielded products of 200, 200/100/100 and 100 bp corresponding to the Ser/Ser, Ser/Cys and Cys/Cys genotypes of the OGG1 gene, respectively. The cleavage with *Pst*I produced fragments of 161, 161/120/41 and 120/41 bp corresponding to the Lys/Lys, Lys/Gln and Gln/Gln genotypes of the *ERCC2* gene, respectively.

**Table 2:** Primers used to analyze p.Arg194Trp, p.Arg399Gln polymorphisms of the *XRCC1* gene, p.Ser326Cys polymorphism of the *hOGG1* and p.Lys751Gln polymorphism of the *ERCC2* gene

Gene	Polymorphism	Primers
*XRCC1*	p.Arg194Trp	forward 5’-GCCCGTCCCAGGTA-3’
		reverse 5’-AGCCCCAAGACCCTTTCACT-3’
	p.Arg399Gln	forward 5’-CAAGTACAGCCAGGTCCTAG-3’
		reverse 5’-CCTTCCCTCTGGAGTAC-3’
*hOGG1*	p.Ser326Cys	forward 5’-GGAAGGTGCTTGGGGAAT-3’
		reverse 5’-ACTGTCACTAGTCTCACCAG-3’
*ERCC2*	p.Lys751Gln	forward 5’-CTGCTCAGCCTGGAGCAGC-3’
		reverse 5’-TAGAATCAGAGAGAGGAGACGCTG-3’.

### Data Analysis

Logistic regression analysis was used to compute odds ratio (OR) and associated 95 % confidence interval (95 % CI) relating each of the SNPs as well as combinations of SNPs and another analysed factors presented in Table 1 to the risk of EC. Only matching variables and factors that altered the ORs by 10 % were included in the final multivariate models. Analyses were performed using STATISTICA 10 package (Statsoft, Tulsa, OK, USA).

## Results

All distributions of genotypes did not differ significantly (*p* < 0.05) from those expected by the Hardy-Weinberg equilibrium. An association (OR 3.95; 95 % CI 1.88–8.31) was found between the Gln/Gln genotype of the p.Lys751Gln polymorphism of *ERCC2* gene and EC occurrence (Table 3). There were no differences in the genotype distributions between cancer patients and controls for the remaining polymorphisms (Tables 4, 5 and 6). We also analyzed combined genotype of all polymorphism pairs. The Arg/Arg genotype of the *XRCC1* gene increased the risk of EC for the carriers of the 751 Gln/Gln variant of the *ERCC2* gene (Table 7). We also found that the Cys/Cys and Arg/Arg genotypes of the p.Ser326Cys polymorphism of the *hOGG1* gene and the Arg/Gln genotype of the *XRCC1* gene decreased EC risk (OR 0.50; 95 % CI 0.25–0.99) (Table 8). No difference between genotype distributions was found for others combined genotypes of the polymorphisms (data not shown). Adjustment for first degree relatives cancer history increased OR for the Gln/Gln genotype of the p.Lys751Gln polymorphism of *ERCC2* gene from OR 3.95; 95 % CI 1.88–8.31 to OR 4.97; 95 % CI 1.98–12.48. Other remaining confounders, including postmenopausal hormone use and body mass index, did not modify the observed estimates of association.

**Table 3:** The allele and genotype frequency and odds ratio (OR) of p.Lys751Gln polymorphism of the *ERCC2* gene in endometrial cancer

	Patients (*n* = 94)		Controls (*n* = 114)		
Genotype or Allele	Number	Frequency	Number	Frequency	OR (95 % CI)
Lys/Lys	30	0.32	38	0.33	0.93 (0.52–1.67)
Lys/Gln	36	0.38	64	0.56	0.48 (0.27–0.84)
Gln/Gln	28	0.30	12	0.11	3.95 (1.88–8,31)
Lys	96	0.52	140	0.61	0.65 (0.44–0.96)
Gln	92	0.48	88	0.39	1.52 (1.03–2.25)

**Table 4:** The allele and genotype frequency and odds ratio (OR) of the p.Ser326Cys polymorphism of the *hOGG1* gene in endometrial cancer

	Patients (*n* = 94)		Controls (*n* = 114)		
Genotype or Allele	Number	Frequency	Number	Frequency	OR (95 % CI)
Ser/Ser	64	0.68	83	0.73	0.79 (0.43–1.44)
Ser/Cys	23	0.24	28	0.24	0.99 (0.52–1.87)
Cys/Cys	7	0.07	3	0.02	2.97 (0.74–11.84)
Ser	151	0.82	194	0.85	0.71 (0.42–1.19)
Cys	37	0.18	34	0.15	1.39 (0.83–2.33)

**Table 5:** The allele and genotype frequency and odds ratio (OR) of the p.Arg399Gln polymorphism of the *XRCC1* gene in endometrial cancer

	Patients (*n* = 94)		Controls (*n* = 114)		
Genotype or Allele	Number	Frequency	Number	Frequency	OR (95 % CI)
Arg/Arg	27	0.29	43	0.37	0.66 (0.37–1.19)
Arg/Gln	45	0.48	48	0.42	1.22 (0.72–2.18)
Gln/Gln	22	0.23	23	0.21	1.21 (0.62–2.34)
Arg	99	0.53	134	0.58	0.78 (0.52–1.15)
Gln	89	0.47	94	0.42	1.28 (0.86–1.89)

**Table 6:** The allele and genotype frequency and odds ratio (OR) of the p.Arg194Trp polymorphism of the *XRCC1* gene in endometrial cancer

	Patients (*n* = 94)		Controls (*n* = 114)		
Genotype or Allele	Number	Frequency	Number	Frequency	OR (95 % CI)
Arg/Arg	89	0.95	103	0.90	1.90 (0.64–5.67)
Arg/Trp	5	0.05	11	0.10	0.53 (0.17–1.57)
Trp/Trp	0	–	0	–	–
Arg	183	0.97	217	0.95	1.85 (0.63–5.43)
Trp	5	0.03	11	0.05	0.50 (0.18–1.57)

**Table 7:** The distribution of combined genotypes of the of the p.Arg194Trp polymorphism of the *XRCC1* gene and p.Lys571Gln polymorphism of the *ERCC2* gene in endometrial cancer

	Patients (*n* = 94)		Controls (*n* = 114)		
Genotype or Allele	Number	Frequency	Number	Frequency	OR (95 % CI)
Arg/Arg – Lys/Lys	28	0.30	36	0.31	0.92 (0.51–1.66)
Arg/Arg – Lys/Gln	33	0.35	57	0.50	0.54 (0.31–0.95)
Arg/Arg – Gln/Gln	28	0.30	10	0.09	4.41 (2.01–9.67)
Arg/Trp – Lys/Lys	2	0.02	2	0.02	1.21 (0.16–8.81)
Arg/Trp – Lys/Gln	3	0.03	7	0.06	0.49 (0.12–1.96)
Arg/Trp – Gln/Gln	0	–	2	0.02	–
Trp/Trp – Lys/Lys	0	–	0	–	–
Trp/Trp – Lys/Gln	0	–	0	–	–
Trp/Trp – Gln/Gln	0	–	0	–	–

**Table 8:** The distribution of combined genotypes of the p.Ser326Cys polymorphism of the *hOGG1* gene and p.Arg399Gln polymorphism of the *XRCC1* gene in endometrial cancer

	Patients (*n* = 94)		Controls (*n* = 114)		
Genotype or Allele	Number	Frequency	Number	Frequency	OR (95 % CI)
Ser/Ser – Arg/Arg	16	0.17	33	0.29	0.50 (0.25–0.99)
Ser/Ser – Arg/Gln	30	0.32	34	0.30	1.10 (0.61–1.99)
Ser/Ser – Gln/Gln	18	0.19	16	0.14	1.45 (0.69–3.01)
Ser/Cys – Arg/Arg	9	0.10	9	0.08	1.23 (0.47–3.25)
Ser/Cys – Arg/Gln	12	0.13	12	0.10	1.24 (0.53–2.91)
Ser/Cys – Gln/Gln	2	0.02	7	0.06	0.33 (0.06–1.63)
Cys/Cys – Arg/Arg	2	0.02	1	0.01	2.45 (0.21–27.52)
Cys/Cys – Arg/Gln	3	0.03	2	0.02	1.85 (0.32–11.28)
Cys/Cys – Gln/Gln	2	0.02	0	–	–

## Discussion

In the present study we genotyped four common polymorphisms of the *XRCC1*, *hOGG1* and *ERCC2* DNA repair genes and tested the association between the distributions of their genotypes with EC. These polymorphisms have been shown to have functional significance and may be in part responsible, for the inter-individual difference in capacity of DNA repair in the general population and for low DNA repair efficacy in cancer patients [5–7, 14–17]. We obtained a significantly higher OR than for other analyzed polymorphisms, odds ratio for the Gln/Gln genotype of the p.Lys751Gln polymorphism of the *ERCC2* gene than for genotypes of remaining polymorphisms. The protein encoded by the *ERCC2* gene is involved in transcription-coupled NER and is an important member of the basal transcription factor TFIIH. Exchange of 751 Lys for Gln in the ERCC2 can lead to a conformational change in the encoded protein at the domain of the interaction between ERCC2 and its helicase activator, p44, inside the TFIIH complex [18]. The Gln/Gln variant of the *ERCC2* gene has been associated with an increased risk of lung cancer [10, 11], and correlated with higher risk of skin, bladder and breast cancer [12, 19, 20]. Surprisingly, this polymorphism has been also linked with non-cancer diseases, such as cataract [21]. To date, none studies have addressed the association between alterations in this region of the *ERCC2* gene and EC. Because a proper functioning of the *ERCC2* gene is important for the genomic stability, its alternations may be associated with a higher cancer susceptibility.

Type I EC are estrogen-related. The mechanisms by which estrogens might cause the development of EC remain unclear. Estrogens have the unique chemical structure that distinguish them from other groups of hormones and their metabolism in eukaryotic cells include formation of a variety of intermediate forms and production of ROS. Estrogens undertake oxidative metabolism through hydroxylation pathway, but the major intermediates are 2-OH and 4-OH estrogens [22]. These chemicals are further oxidized to semiquinones and quinones, which may form bulky DNA adducts and may undergo redox cycling, producing ROS that may cause oxidative stress, lipid peroxidation, and DNA damage [23, 24]. Consequently, estrogen metabolism in human cells may play a role in tumor initiation via direct damage to the DNA by the formation of bulky DNA adducts and/or by producing ROS that cause oxidative DNA damage. These types of DNA damage are usually repaired by NER and BER.

In our study we analyzed the association between three polymorphisms of two genes of BER and EC. We did not find any association when we analyzed each polymorphism separately, but the analysis of combined genotypes showed that they might significantly increase the risk of EC. The results obtained suggest that polymorphisms of the *XRCC1* and *ERCC2* genes may modulate the risk and therefore play a role in the etiology of EC. The XRCC1 protein has no known catalytic activity but serves to orchestrate BER through its role as a central scaffolding protein for DNA ligase III, DNA polymerase β, and poly(ADP-ribose) polymerase (PARP) [25]. Arg/Trp variant of the p.Arg/Trp polymorphism of the XRCC1 gene occurs in proliferating cell nuclear antigen binding region, but few studies have examined the influence of the Trp/Trp genotype of this polymorphism on the function of the XRCC1 protein [26, 27]. This variant has been associated with a lower bleomycin and benzo(a)pyrene diol-epoxide sensitivity in vitro [16, 28]. These data suggest a protective role of the Trp/Trp genotype of the p.Arg/Trp polymorphism of the *XRCC1* gene against the development of cancer and this function can be underlined by increasing the activity of BER. This is in agreement with our result suggesting a potential role of the Arg/Arg genotype of the p.Arg/Trp polymorphism of the *XRCC1* gene with reduced BER capacity as compared with Trp/Trp genotype in EC.

We have also found that cancer history in first degree relatives increased endometrial cancer risk in the Gln/Gln variant of the p.Lys751Gln polymorphism of the *ERCC2* gene. This result may suggest hereditary background of EC cancer and/or major contribution of the p.Lys751Gln polymorphism of the *ERCC2* gene in cancer development but more studies performed on larger population is needed to draw a final conclusion.

In summary, our results suggest that the 751 Gln/Gln variant of the p.Lys751Gln polymorphism of the *ERCC2* gene can be associated with the occurrence of EC. We have also showed that the Arg/Arg variant of the p. Arg194Trp polymorphism of the *XRCC1* gene increased the risk of EC in individuals with the Gln/Gln variant of the ERCC2 gene. The data obtained suggest also that positive cancer history in first degree relatives in connection with Gln/Gln variant of the p.Lys751Gln polymorphism of the *ERCC2* gene may be associated with EC.
